# Acquired Obesity Is Associated with Changes in the Serum Lipidomic Profile Independent of Genetic Effects – A Monozygotic Twin Study

**DOI:** 10.1371/journal.pone.0000218

**Published:** 2007-02-14

**Authors:** Kirsi H. Pietiläinen, Marko Sysi-Aho, Aila Rissanen, Tuulikki Seppänen-Laakso, Hannele Yki-Järvinen, Jaakko Kaprio, Matej Orešič

**Affiliations:** 1 Obesity Research Unit, Department of Psychiatry, Helsinki University Central Hospital, Helsinki, Finland; 2 Department of Medicine, Division of Diabetes, Helsinki University Central Hospital, Helsinki, Finland; 3 Finnish Twin Cohort Study, Department of Public Health, University of Helsinki, Helsinki, Finland; 4 VTT Technical Research Centre of Finland, Espoo, Finland; 5 Department of Mental Health and Alcohol Research, National Public Health Institute, Helsinki, Finland; University of Parma, Italy

## Abstract

Both genetic and environmental factors are involved in the etiology of obesity and the associated lipid disturbances. We determined whether acquired obesity is associated with changes in global serum lipid profiles independent of genetic factors in young adult monozygotic (MZ) twins. 14 healthy MZ pairs discordant for obesity (10 to 25 kg weight difference) and ten weight concordant control pairs aged 24–27 years were identified from a large population-based study. Insulin sensitivity was assessed by the euglycemic clamp technique, and body composition by DEXA (% body fat) and by MRI (subcutaneous and intra-abdominal fat). Global characterization of lipid molecular species in serum was performed by a lipidomics strategy using liquid chromatography coupled to mass spectrometry. Obesity, independent of genetic influences, was primarily related to increases in lysophosphatidylcholines, lipids found in proinflammatory and proatherogenic conditions and to decreases in ether phospholipids, which are known to have antioxidant properties. These lipid changes were associated with insulin resistance, a pathogonomic characteristic of acquired obesity in these young adult twins. Our results show that obesity, already in its early stages and independent of genetic influences, is associated with deleterious alterations in the lipid metabolism known to facilitate atherogenesis, inflammation and insulin resistance.

## Introduction

Obesity increases the risk of cardiovascular diseases and diabetes [1] especially when the extra fat is accumulated to central and intra-abdominal depots [2], [3]. The increased cardiometabolic risk in obesity is at least partly mediated through atherogenic dyslipidemia characterized by an increase in plasma triglycerides, large very low density lipoprotein (VLDL) particles, small dense low density lipoprotein (LDL) particles as well as low concentrations of high density (HDL) cholesterol [4]. It is also recognized that changes in the function of individual lipids due to peroxidation, imbalanced fatty acid composition or their altered flux from peripheral tissues may contribute to development of atherosclerosis and diabetes [5].

The advent of novel analytical and information technologies for handling large volumes of data has made it feasible to characterize complex mixtures of lipids in body fluids and tissues and relate them to other entities of biological systems [5], [6]. Therefore, lipidomics as a branch of metabolomics may provide powerful tools for characterization of global lipid profiles and identification of previously unknown changes in lipid metabolism in complex phenotypes such as those related to obesity [7]–[9].

The origin of obesity and related dyslipidemias is multifactorial [10]. Not all obese individuals develop dyslipidemia and not all dyslipidemic patients are obese. While environmental and lifestyle factors play a key role in the development of obesity, genetic variation may determine an individual's susceptibility to body fat accumulation and lipid disturbances [10]. Cross-sectional studies comparing lipid profiles in obese *vs.* non-obese humans do not permit unequivocal distinction between genetic versus environmental and life-style effects. This can best be done by studying monozygotic (MZ) twins discordant for obesity. MZ twins are genetically identical at the sequence level and any differences between the co-twins are thus attributable to environmental factors [11]. Environmental factors are here considered very broadly, including i.e. possible prenatal exposures and effects inducing epigenetic changes. The co-twin design controls for age, gender, childhood socioeconomic background and other environmental experiences and exposures.

In a previous study of young adult obesity-discordant but otherwise healthy MZ twin pairs we have shown that the degree of obesity correlates with increases in visceral and liver fat deposition [12]. These adverse effects were associated with abnormalities in expression of fatty acid transport proteins [13] and increased expression of inflammation markers in the adipose tissue [14], [15].

We then hypothesized that obesity-discordant twins would differ for the levels of specific serum lipid molecular species, and that these lipid species may provide further clues about the early mechanisms and biomarkers associated with acquired obesity and related complications. In this paper we report the results of lipidomics analyses of 14 pairs of MZ twins highly discordant for obesity. We show that acquired obesity primarily relates to increases in lysophosphatidylcholines, constituents of an atherogenic lipid profile and decreases in ether phospholipids, lipids with anti-oxidative properties. Sphingomyelins with long chain fatty acids remain very similar in the weight-discordant co-twins.

## Methods

### Subjects

The participants were recruited from a population-based longitudinal survey of five consecutive birth cohorts (1975–1979) of twins, their siblings and parents, identified through the national population registry of Finland [16]. Twin pairs included in the current study were enrolled based on their responses to questions on weight and height at age 23–27 years [12]–[15], [17], [18]. After screening of all MZ twin pairs (N = 658), we identified 18 pairs with a reported body mass index (BMI) difference of at least four kg/m^2^, such that one co-twin was non-obese (BMI approximately 25 kg/m^2^), while the other was obese (BMI approximately 30 kg/m^2^). Fourteen of these pairs (eight male and six female pairs) participated in the present study. We also studied ten randomly selected weight-concordant MZ pairs (reported BMI difference of less than two kg/m^2^, two male and three female over-weight pairs, three male and two female normal-weight pairs). The measured BMI differences ranged from 3.3 to 10.1 kg/m^2^ in the discordant and from 0.0 to 2.3 kg/m^2^ in the concordant pairs. Discordant pairs were of the same age (mean±SD 25.6±1.2 years) as the concordant pairs (25.7±1.2 years).

The subjects were healthy (based on medical history and clinical examination by a physician (KHP), normotensive and did not use any medications except contraceptives. Their psychiatric health was confirmed by a structured psychiatric interview ‘Semi-structured assessment for the genetics of alcohol interview instrument’ (SSAGA) [19], supplemented by the eating disorders sections of the Structured Clinical Interview for DSM-IV-TR (SCID). The SSAGA provides diagnoses of common psychiatric conditions including depression and substance abuse and dependence based on Diagnostic and Statistical Manual of Mental Disorders (DSM) and International Classification of Diseases (ICD) diagnostic criteria. The twins were interviewed by telephone by two experienced interviewers, such that each member of twin pair was interviewed by a different interviewer; the interviewers were blind as to the clinical details and weight status of the twin.

All twins had been weight-stable for at least 3 months prior to the study. Females were scheduled to attend during the follicular phase of their menstrual cycle. All the concordant pairs were nonsmokers, but four out of 14 obesity-discordant pairs were discordant for smoking. In three pairs, the one who smoked more was leaner, but in one pair, the co-twin who smoked more was heavier. Monozygosity was confirmed by genotyping of ten informative genetic markers [17]. The subjects provided written informed consent. The protocol was designed and performed according to the principles of the Helsinki Declaration and was approved by the Ethical Committee of the Helsinki University Central Hospital.

### Clinical assessments

All subjects were studied after an overnight fast starting at 8 a.m. Five ml venous blood samples were obtained from which plasma with ethylenediaminetetraacetate (EDTA) and serum were separated by centrifugation, frozen immediately at −20°C, moved to −80°C within 6 hours and stored until analysis. Body composition was measured by dual-energy x-ray absorptiometry (DEXA) (Lunar Prodigy, Madison, WI, software version 2.15) [20]. Subcutaneous (SC) and intra-abdominal (IA) fat were determined by magnetic resonance imaging (MRI) of 16 transaxial scans reaching from 8 cm above to 8 cm below the fourth and fifth lumbar interspace [12]. Three day food diaries completed by the subjects were reviewed by a trained nutritionist, and analyzed by using the program DIET32, which is based on a national database for food composition (Fineli) (http://www.ktl.fi/fineli/).

### Insulin sensitivity

Whole body insulin sensitivity was determined by the euglycemic hyperinsulinemic clamp technique [21]. Two 18-gauge catheters (Venflon; Viggo-Spectramed, Helsingborg, Sweden) were inserted, one in an antecubital vein for infusion of insulin and glucose, and another retrogradely in a heated hand vein to obtain arterialised venous blood for measurement of glucose concentrations every 5 min and serum free insulin concentration every 30 min. Regular human insulin (Insulin Actrapid; Novo Nordisk, Denmark) was infused in a primed-continuous fashion. The rate of the continuous insulin infusion was 40 mU/m^2^·min (1 mU/kg⋅min) for 120 min. Normoglycemia was maintained by adjusting the rate of a 20% glucose infusion based on plasma glucose measurements from arterialised venous blood every 5 min. Whole body insulin sensitivity (the M-value, expressed as mg/kg fat free mass per minute) was determined from the glucose infusion rate needed to maintain normoglycemia after correction for changes in the glucose pool size [21]. Since hepatic glucose production is maximally suppressed in non-diabetic subjects already at an insulin concentration achieved during infusion of insulin at a rate of 0.5 mU/kg⋅min [22], the M-value mostly reflects glucose uptake. Plasma glucose concentrations were measured in duplicate with the glucose oxidase method (Glucose Analyzer II; Beckman Instruments, Fullerton, CA, USA) [23]. Serum free insulin concentrations were determined with radioimmunoassay (Phadeseph Insulin RIA, Pharmacia & Upjohn Diagnostics, Uppsala, Sweden) after precipitation with polyethylene glycol [24].

### Clinical chemistry

Serum total, high density lipoprotein cholesterol (HDL) and triglyceride concentrations were measured with respective enzymatic kits from Roche Diagnostics using an autoanalyzer (Roche Diagnostics Hitachi 917, Hitachi Ltd, Tokyo, Japan). Low density lipoprotein cholesterol (LDL) concentrations were calculated using the formula of Friedewald [25]. Serum leptin concentrations were measured using enzyme-linked immunoassays (Quantikine R&D Systems, Minneapolis, MN, USA) and adiponectin concentrations using the ELISA kit from B-Bridge International (San Jose, CA, USA). High-sensitivity C-reactive protein (CRP) was measured using a sensitive double antibody sandwich ELISA with rabbit antihuman CRP and peroxidase conjugated rabbit anti-human CRP.

### Lipid nomenclature

Lipids from the lipidomic analysis were named according to Lipid Maps (http://www.lipidmaps.org) [26]. For example, lysophosphatidylcholine with 16∶0 fatty acid chain was named as monoacyl-glycerophosphocholine GPCho(16∶0/0∶0). In case the fatty acid composition was not determined, total number of carbons and double bonds was marked. For example, a phosphatidylcholine species GPCho(16∶0/20∶4) is represented as GPCho(36∶4). However, GPCho(36∶4) could also represent other molecular species, for example GPCho(20∶4/16∶0) or GPCho(18∶2/18∶2).

### Lipidomics analysis

**Figure 1:**
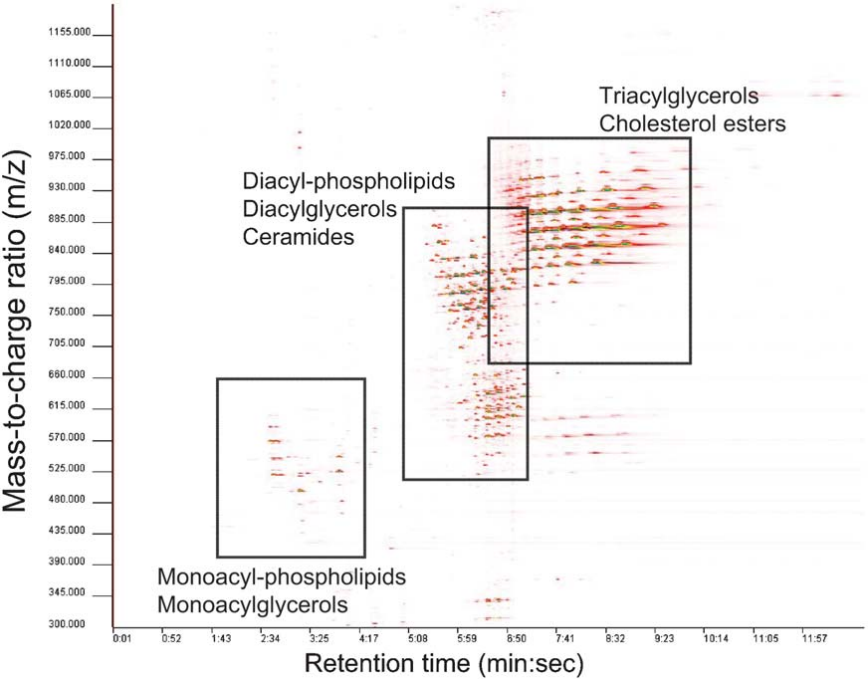
Two-dimensional representation of the lipid spectra for a selected illustrative sample obtained by reversed phase Ultra Performance Liquid Chromatography coupled to high resolution Mass Spectrometry (positive ion mode). Major lipid groups found in different parts of the spectra are marked. MZmine software [18], [19] was utilized for visualization and spectral data processing.

To examine the lipid phenotypes of the twin pairs, the Ultra Performance Liquid Chromatography Mass Spectrometry (UPLC/MS) based lipidomics analysis was performed. The applied platform affords broad screening of multiple lipid classes from total lipid extracts within a single sample run. With UPLC, a run time of 12 minutes can be achieved without significant loss in sensitivity, covering major monoacyl-glycerols and -phospholipids, diacyl-glycerols and –glycerophospholipids, sphingolipids, triacylglycerols, and cholesterol esters (Figure 1). The lipid analysis is the same as described in detail previously [27].

An aliquot (10 µl) of an internal standard mixture containing equal amounts of, internal standards (GPCho(17∶0/0∶0), GPCho(17∶0/17∶0), GPEtn(17∶0/17∶0), GPGro(17∶0/17∶0)[rac], Cer(d18∶1/17∶0), GPSer(17∶0/17∶0), GPA(17∶0/17∶0) and D-*erythro*-Sphingosine-1-Phosphate (C17 Base) from Avanti Polar Lipids and MG(17∶0/0∶0/0∶0)[rac], DG(17∶0/17∶0/0∶0)[rac] and TG(17∶0/17∶0/17∶0) from Larodan Fine Chemical) and 0.05 M sodium chloride (10 µl) were added to serum samples (10 µl) and the lipids were extracted with chloroform/methanol (2∶1, 100 µl). After vortexing (2 min), standing (1 hour) and centrifugation (10000 RPM, 3 min), the lower layer was separated and a standard mixture containing 3 labeled standard lipids was added (10 µl) to the extracts. The standard solution contained 10 µg/ml (in chloroform:methanol 2∶1) GPCho(16∶0/0∶0-D3), GPCho(16∶0/16∶0-D6) and TG(16∶0/16∶0/16∶0-^13^C3), all from Larodan Fine Chemicals. The sample order for LC/MS analysis was determined by randomization.

Lipid extracts were analysed on a Waters Q-Tof Premier mass spectrometer combined with an Acquity Ultra Performance LC™. The column, which was kept at 50°C, was an Acquity UPLC^TM^ BEH C18 10×50 mm with 1.7 µm particles. The binary solvent system included A. water (1% 1 M NH_4_Ac, 0.1% HCOOH) and B. LC/MS grade (Rathburn) acetonitrile/isopropanol (5∶2, 1% 1 M NH_4_Ac, 0.1% HCOOH). The gradient started from 65% A/35% B, reached 100% B in 6 min and remained there for the next 7 min. The total run time including a 5 min re-equilibration step was 18 min. The flow rate was 0.200 ml/min and the injected amount 0.75 µl. The temperature of the sample organizer was set at 10°C.

The lipid profiling was carried out on Waters Q-Tof Premier mass spectrometer using ESI+ mode. The data were collected at mass range of m/z 300–1200 with a scan duration of 0.2 sec. The source temperature was set at 120°C and nitrogen was used as desolvation gas (800 L/h) at 250°C. The voltages of the sampling cone and capillary were 39 V and 3.2 kV, respectively. Reserpine (50 µg/L) was used as the lock spray reference compound (5 µl/min; 10 sec scan frequency).

Tandem mass spectrometry was used for the identification of selected molecular species of lipids. MS/MS runs were performed by using ESI+ mode and ESI- mode (to determine fatty acid composition of phospholipids), collision energy ramp from 15 to 30 V and mass range starting from m/z 150. The other conditions were as shown above.

Data were processed using MZmine software version 0.60 [28]. Lipids were identified using internal spectral library or with tandem mass spectrometry. The normalization of lipidomics data was performed as follows: All monoacyl lipids except cholesterol esters, such as monoacylglycerols and monoacyl-glycerophospholipids were normalized with GPCho(17∶0/0∶0), all diacyl lipids except ethanolamine phospholipids were normalized with GPCho(17∶0/17∶0), the diacyl ethanolamine phospholipids were normalized with GPEtn(17∶0/17∶0), and the triacylglycerols and cholesterol esters with TG(17∶0/17∶0/17∶0). Only the identified lipid molecular species were included in data analyses, unless otherwise noted.

### Chemometric modeling of data

Partial least squares discriminant analysis (PLS/DA) [29], [30] was utilized as a supervised modeling method using SIMPLS algorithm to calculate the model [31]. As the total number of samples was insufficient for independent validation, no hold-out dataset was utilized for cross-validation. Instead, Venetian blinds cross-validation method [32] and *Q* ^2^ scores were used to optimize the model. Top loadings for latent variables were reported. The VIP (variable importance in the projection) values [33] were calculated to identify the most important molecular species for the clustering of specific groups. Multivariate analyses were performed using Matlab version 7.2 (Mathworks, Inc.) and the PLS Toolbox version 4.0 Matlab package (Eigenvector Research, Inc.).

### Statistical analyses

The statistical comparisons of the subjects were made either between individuals or between co-twins in a pair. Given that MZ twins are genetically identical, any differences between the co-twins must be attributable to acquired factors, including prenatal and epigenetic effects [11]. Differences in the clinical characteristics between the co-twins were compared by paired Wilcoxon's signed ranks test. Male and female pairs were combined because MZ co-twins are inherently matched for gender. To analyze the relationships between lipidomics and clinical measures independent of potential genetic confounding, the data were twin-normalized in all pairs.

Previous studies have shown that the variance of intensities tends to grow linearly as a function of the intensity levels (heteroscedasticity) [34]. By taking logarithms of the intensity values one can eliminate this effect and make the data better follow a normal distribution with intensity-independent variance. For this reason, the body composition data were twin-normalized by using the difference between the heavier and leaner co-twins (based on BMI) and the biochemical variables by using the difference of log2-transformed values. Lipidomics data were twin-normalized by taking the within-pair difference of the log2-transformed concentration values for each lipid species:
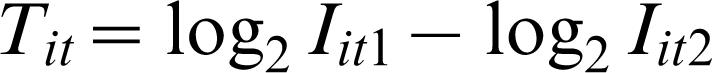
where *T_it_* is the twin-normalized lipid intensity, *i* is the lipid index, *t* is the twin pair index (1 denoting the twin with higher BMI, 2 with lower), and *I_it_*_1_ is the peak height for lipid *i* of the heavier twin from pair *t*. Within-pair similarity was assessed for each lipid molecular species by a total sum of squares of log differences of lipid levels within each twin pair, i.e.
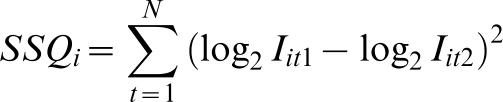



The correlations between the clinical variables and the lipid profiles were calculated pair-wise using Spearman rank correlations. The reported *p*-value is the probability of getting a correlation as large as the observed value by random chance, when the true correlation is zero. The *p*-value was calculated using the large sample approximation. The levels of significance are marked as *p<0.05, **p<0.01, ***p<0.001. Only correlations with *p*<0.15 were reported in figures, unless noted otherwise. Partial Least Squares method was also applied to regress the lipid profiles on the clinical variables. Since the results were similar and conclusions the same as obtained by pair-wise correlation analysis, these results are not reported.

## Results

### Clinical characteristics of the co-twins

Physical and biochemical characteristics of the twins are shown in Table 1. The obese co-twins of the discordant MZ pairs had more subcutaneous and intra-abdominal fat than the non-obese co-twins. The obese co-twins also had lower whole body insulin sensitivity (M-value) and higher fasting serum insulin concentrations than the non-obese co-twins. Fasting serum LDL-cholesterol and triglyceride concentrations were higher and HDL-cholesterol concentrations lower in the obese co-twins. Fasting serum CRP and leptin concentrations were higher and adiponectin concentrations lower in the obese, compared with the non-obese co-twins. None of these variables differed between the weight-concordant co-twins (Table 1). The two groups of non-obese individuals (*i.e.* the non-obese discordant and the normal weight concordant pairs) differed from each other. As shown in Table 1, the normal weight concordant pairs were leaner than the non-obese discordant twins, having lower BMI (*p* = 0.007), % body fat (*p* = 0.033), subcutaneous (*p* = 0.012) but not intra-abdominal fat (*p* = 0.31), and lower CRP (*p* = 0.013) and leptin (*p* = 0.027). However, the main interest in this study was in the within pair comparisons of the pairs. Compared with the concordant pairs, the discordant pairs had larger intra-pair differences in BMI (median 5.1 *vs*. 1.0 kg/m^2^, *p*<0.0001), % body fat (7.3 *vs*. 2.4%, *p* = 0.0002), subcutaneous fat (1784 *vs*. 254 cm^3^, *p* = 0.0007), intra-abdominal fat (375 *vs*. 93 cm^3^, *p* = 0.0049), the M-value (−2.9 *vs*. 0.4 mg·kg fat free mass^−1^·min^−1^, *p* = 0.016), fasting serum insulin (4.0 *vs*. 0.5, *p* = 0.052), leptin (12.0 *vs*. 0.6 ng/ml, *p* = 0.0004) and adiponectin (−16.5 *vs*. 3.8 µg/ml, *p* = 0.041), but not in serum total cholesterol, LDL, HDL, triglycerides or CRP (data not shown). In the following twin-pair normalized analyses, both concordant and discordant pars were used, to yield a wide range of intra-pair differences, from very similar to dissimilar pairs. In the whole sample, the BMI-differences ranged from 0.01 to 10.1 kg/m^2^, and individual BMIs from 20.0 to 45.8 kg/m^2^.

**Table 1:** Physical and biochemical characteristics of the 24 monozygotic twin pairs.

	Pairs discordant for weight (n = 14)			Pairs concordant for weight (n = 10)	
				Normal weight pairs (n = 5)	Overweight pairs (n = 5)
	Non-obese co-twin	Obese co-twin	*p* *	Both co-twins	Both co-twins
BMI (kg/m^2^)	25.4 (24.5, 26.1)	30.4 (28.4, 32.5)	0.001	21.5 (20.8, 23.7)	29.3 (26.9, 30.4)
Percent body fat	27.1 (23.3, 38.5)	37.2 (32.6, 43.0)	0.001	19.0 (13.9, 20.7)	34.8 (29.9, 45.8)
Subcutaneous fat (cm^3^)	2698 (2406, 3966)	4846 (4376, 5821)	0.001	1445 (1234, 1600)	4162 (3675, 5917)
Intra-abdominal fat (cm^3^)	527 (380, 685)	1010 (827, 1183)	0.001	452 (361, 614)	1059 (626, 1500)
M value (mg·kg fat free mass^−1^·min^−1^)	8.6 (6.6, 10.4)	6.6 (4.9, 7.3)	0.01	8.4 (6.2, 9.8)	6.9 (5.7, 8.0)
Serum insulin (mU/l)	5.0 (3.0, 8.0)	9.0 (6.0, 11.0)	0.02	5.5 (4.0, 6.0)	9.5 (7.0, 10.0)
Serum total cholesterol (mmol/l)	4.6 (4.1, 5.0)	4.7 (4.3, 5.1)	0.41	4.4 (3.9, 5.1)	4.7 (4.4, 5.5)
Serum LDL-cholesterol (mmol/l)	2.5 (2.1, 3.0)	2.6 (2.4, 3.2)	0.030	2.5 (1.5, 2.9)	2.7 (2.3, 3.0)
Serum HDL-cholesterol (mmol/l)	1.3 (1.2, 1.7)	1.3 (1.0, 1.4)	0.012	1.4 (1.1, 2.0)	1.2 (1.1, 1.8)
Serum triglycerides (mmol/l)	1.0 (0.7, 1.2)	1.4 (0.9, 1.6)	0.028	1.0 (0.8, 1.1)	1.6 (0.7, 2.1)
Serum high sensitivity CRP (mg/l) a	0.9 (0.2, 1.1)	2.0 (0.9, 3.4)	0.003	0.2 (0.1, 0.2)	1.4 (0.5, 4.0)
Serum leptin (ng/ml) b	15.1 (7.2, 25.3)	24.1 (18.5, 39.1)	0.003	4.8 (3.6, 6.1)	17.7 (11.1, 39.4)
Serum adiponectin (µg/ml) b	12.7 (9.0, 14.9)	10.8 (6.8, 12.6)	0.013	10.7 (9.2, 11.9)	7.9 (6.9, 9.1)

Data are median (interquartile range). ^*^obese vs. non-obese co-twins, paired Wilcoxon's test. ^a^ n = 19 pairs. ^b^ n = 21 pairs.

**Figure 2:**
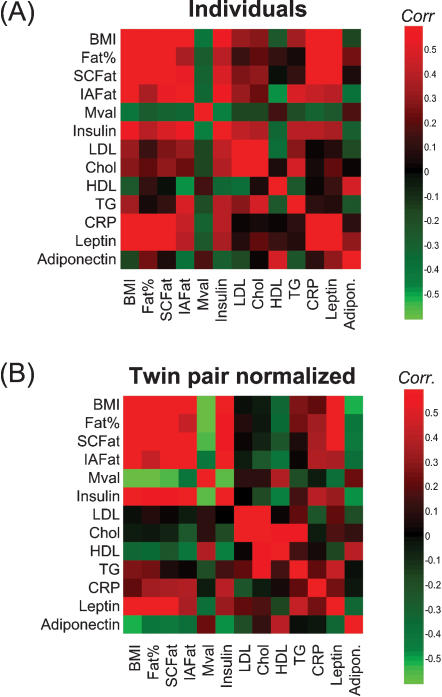
Correlation maps for clinical variables. (A) Each individual considered separately (N = 48). (B) Twin pair normalized (N = 24).

Relationships between the measures of obesity and other clinical characteristics were analyzed for each individual separately and within twin pairs (Figure 2). After twin-pair normalization, i.e. after controlling for genetic factors, the negative correlations of different measures of body fat with whole body insulin sensitivity (the M-value) and adiponectin and positive correlations with fasting serum insulin and leptin became more pronounced than in the correlations performed in individual subjects. In addition, twin-pair normalization also resulted in clearer negative correlations of HDL cholesterol with obesity and with insulin sensitivity than correlations at individual level.

The reported intake of energy and macronutrients was similar in the co-twins of both the discordant and concordant pairs. In the discordant pairs, the obese co-twins reported a median daily consumption of 8554 kJ energy, 78 g fat, 85 g protein and 240 g carbohydrates. In the non-obese co-twins, the respective values were 9281 kJ energy, 80 g fat, 82 g protein and 258 g carbohydrates.

### Lipid profiles and obesity

**Figure 3:**
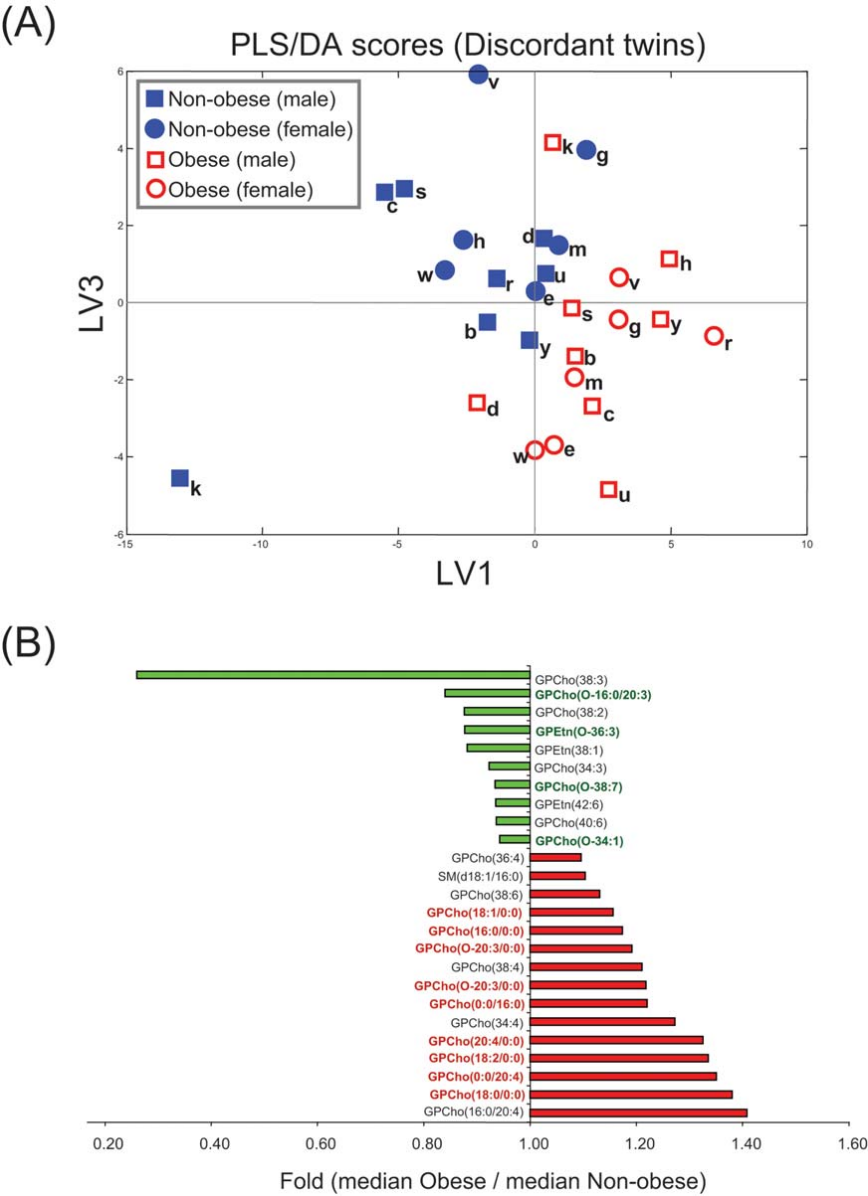
Partial least squares discriminant analysis (PLS/DA) of lipidomics profiles for obesity discordant co-twins, utilizing only the 133 identified peaks and two classes (obese and non-obese co-twins) to build the model. Three latent variables were used (Q^2^ = 47%). (A) PLS/DA score plot. Genders and twin-pair identifiers are marked for each sample, although this information was not used to build the model. (B) Fold changes for most important variables based on VIP analysis contributing to the PLS/DA model.

A total of 331 lipid molecular species were detected, of which 133 were identified (Supplementary Dataset S1). Distinct differences were observed in the global lipid profiles between the obese and non-obese co-twins in the 14 MZ twin pairs discordant for obesity (Figure 3A). While the concentrations of most abundant phospholipids such as phosphatidylcholines were found both increased or decreased depending on their fatty acid composition, two consistent trends were found: concentrations of lysophosphatidylcholines (LPCs) were higher and concentrations of ether phospholipids lower in the obese co-twins (Figure 3B). The observed differences were not explained by gender (Figure 3A).

**Figure 4:**
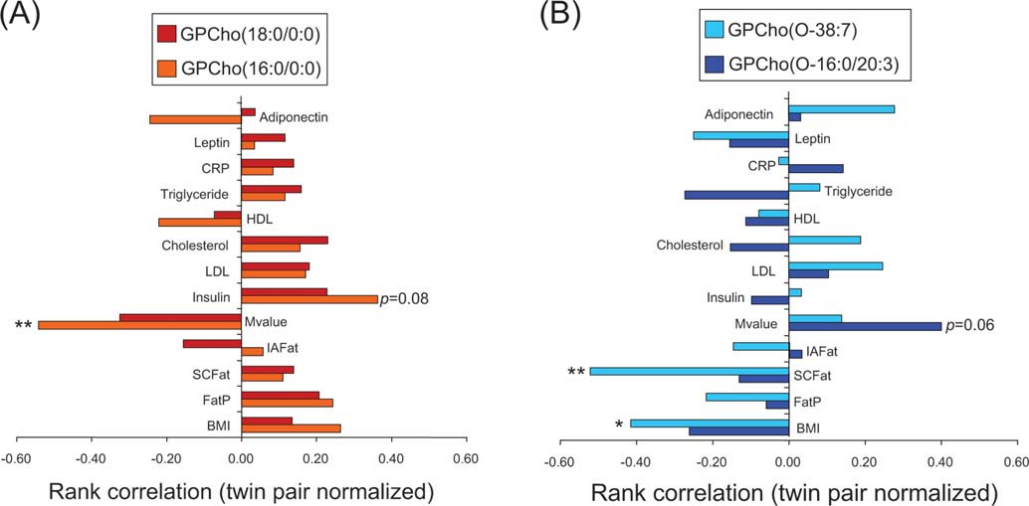
Twin-normalized Spearman rank correlations of (A) two most abundant lysophosphatidylcholine and (B) ether phospholipid species with clinical variables. ^*^ *p*<0.05, ^**^ *p*<0.01.

Pairwise analyses revealed that LPCs correlated positively with measures subcutaneous obesity and negatively with insulin sensitivity (Figure 4A), while the reverse was found for ether phospholipids (Figure 4B). No consistent relationships were observed between LPCs or ether phospholipids and intra-abdominal fat.

**Figure 5:**
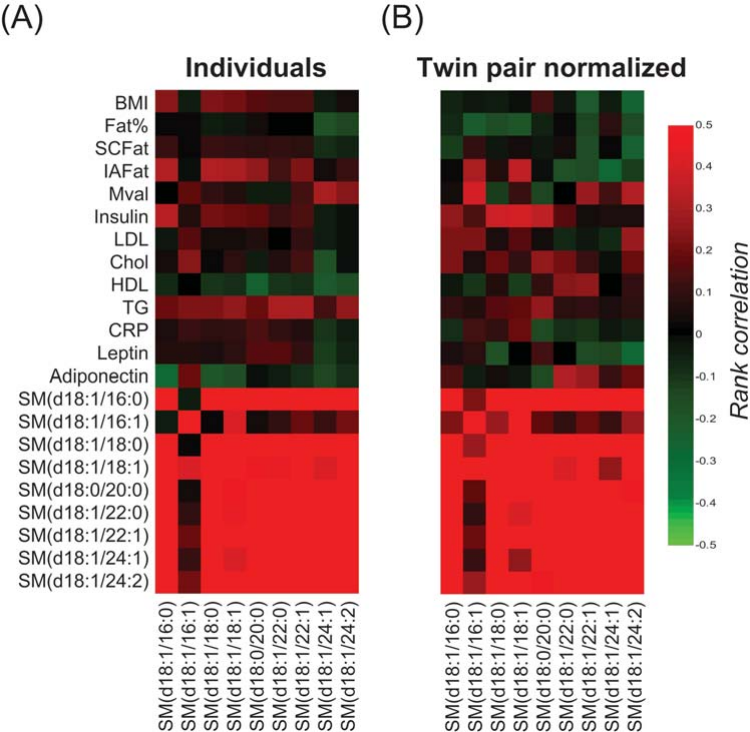
Correlation plots for selected sphingomyelin species and clinical variables in (A) individual twins, and (B) twin pairs.

We also sought for the lipid species with the least intrapair variability (SSQ value). Out of all 331 peaks, the five with the lowest variability were all sphingomyelins (SMs) with very long chain fatty acids (Table 2). These very long chain fatty acid SMs had no correlation with body composition, whereas short and medium chain fatty acid SMs correlated positively with intra-abdominal fat and insulin (Figure 5).

**Table 2:** The most similar lipids within each twin pair (N = 24).

Lipid ID	Mean	SSQ/N
**SM(d18∶0/20∶0)**	0.0145	0.0882
**SM(d18∶1/22∶0)**	−0.0349	0.0980
**SM(d18∶1/22∶1)**	−0.0368	0.0744
**SM(d18∶1/24∶1)**	−0.0363	0.0825
**SM(d18∶1/24∶2)**	−0.0376	0.0948

### Body fat distribution and the lipid profiles

**Figure 6:**
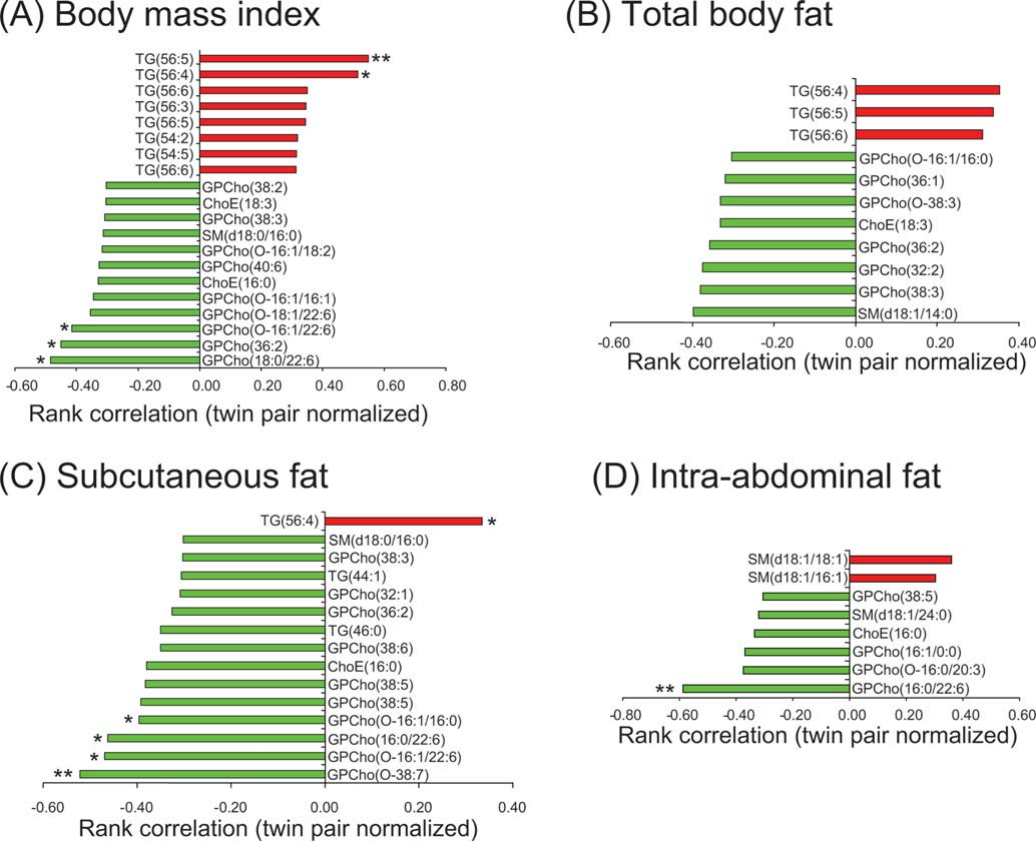
Twin-normalized Spearman rank correlations of lipids with different fat depots: (A) BMI, (B) total body fat, (C) subcutaneous fat, and (D) intra-abdominal fat. ^*^ *p*<0.05, ^**^ *p*<0.01.

We further studied whether serum lipid profiles would differ by various measures of obesity. Within twin pairs, all fat depots were negatively correlated with docosahexaenoic acid (22∶6, commonly known as DHA) containing phospholipids and phosphatidylcholine molecular species GPCho(38∶5) (Figure 6). The measures of subcutaneous fat shared some features that differed from those of intra-abdominal fat. BMI, total body fat and subcutaneous fat correlated positively with long chain triacylglycerol species (≥C54) and negatively with specific phosphatidylcholine species and the ether phospholipids (Figure 6A–C). Intra-abdominal fat correlated positively with medium chain sphingomyelin species (Figure 6D).

### Insulin sensitivity and the lipid profiles

**Figure 7:**
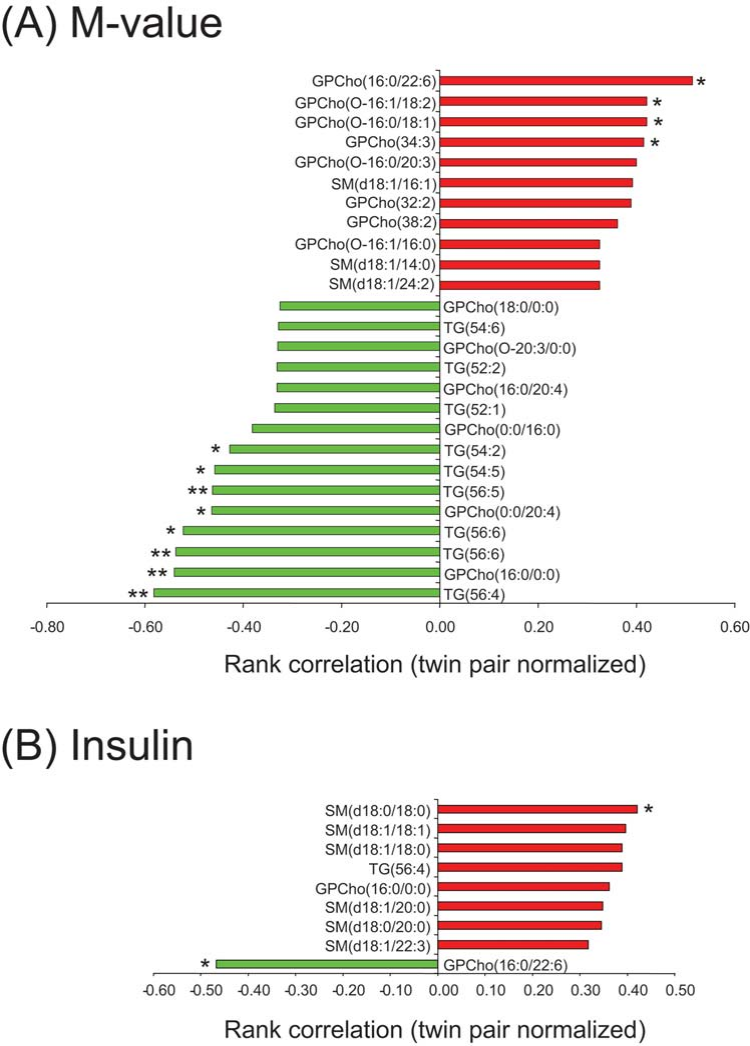
Twin-normalized Spearman rank correlations of lipids with (A) M-value and (B) insulin. ^*^ *p*<0.05, ^**^ *p*<0.01.

Many of the same lipids that were previously shown to correlate positively with measures of subcutaneous fat (Figure 6A–C) were negatively associated with insulin sensitivity (Figure 7A). LPCs and triacylglycerol species containing ≥C54 were associated with poorer and DHA-containing phosphatidylcholines and ether phospholipids with better insulin sensitivity. The same sphingomyelin species SM(d18∶1/18∶1) that correlated positively with intra-abdominal fat (Figure 6D), correlated positively with fasting insulin (Figure 7B). Several other sphingomyelin species containing 18∶0, 18∶1, and 20∶0 fatty acids also correlated positively with insulin.

### Correlations of the global lipid profile with classical lipid parameters

**Figure 8:**
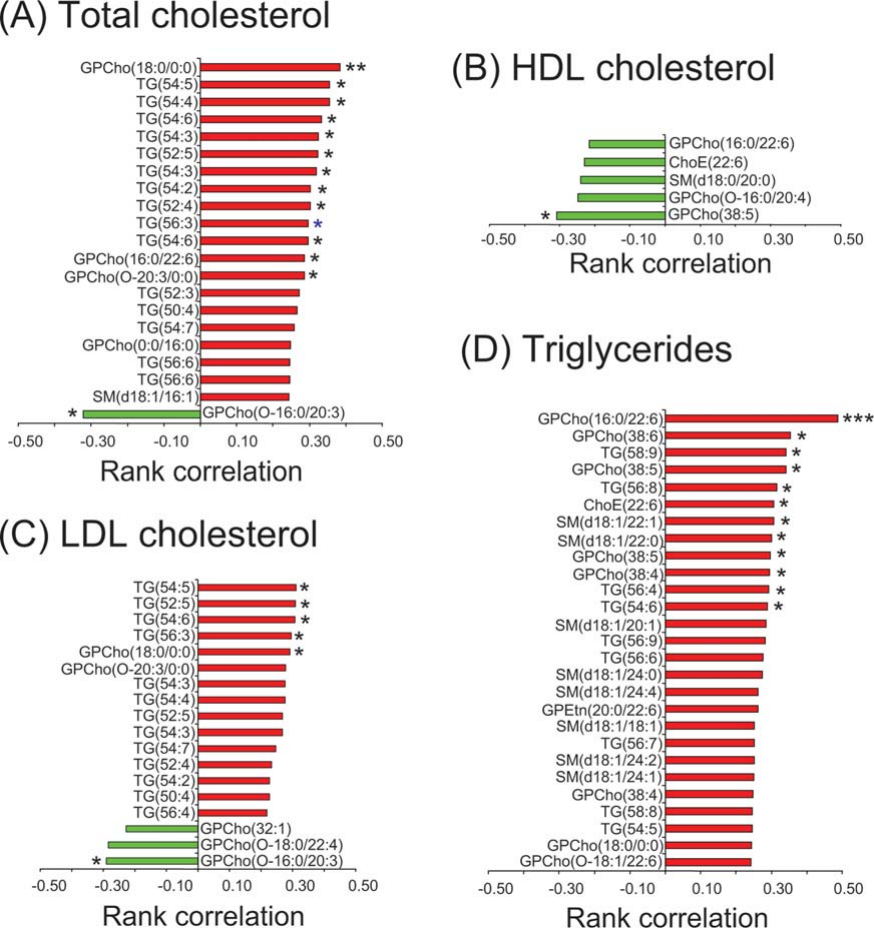
Correlations with classical lipid parameters using Spearman rank correlations across all individuals (*N* = 48). Only lipids with *p*<0.1 are reported for triglyceride and total cholesterol correlations. ^*^ *p*<0.05, ^**^ *p*<0.01, ***p<0.001.

Correlations between lipid molecular species and classical serum lipids were analyzed in individual twins. The total cholesterol concentration correlated positively with medium and long chain triacylglycerols and with specific LPCs, and negatively with the ether phospholipids (Figure 8A). The HDL cholesterol correlated poorly with the lipidomic profiles, with only few molecular species being significantly negatively correlated, none of them triacylglycerols (Figure 8B). The LDL cholesterol had similar correlation structure as the total cholesterol (Figure 8C) while the total triglycerides correlated best to specific phosphatidylcholines, including the DHA-containing GPCho(16∶0/22∶6), long and medium chain triacylglycerols, as well as to long chain fatty acid sphingomyelins (Figure 8D).

**Figure 9:**
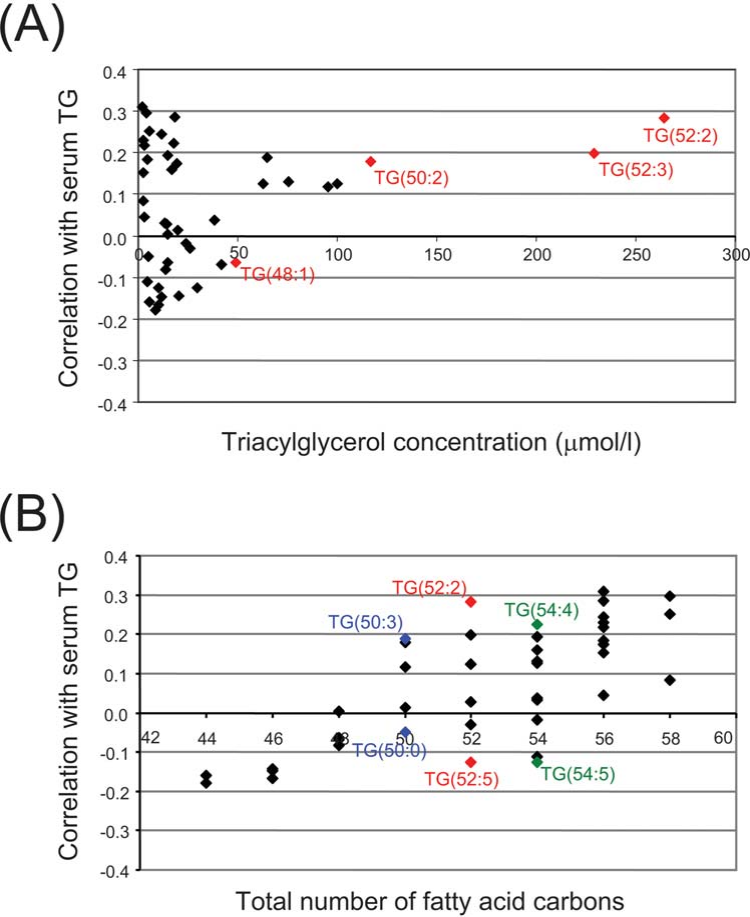
Correlation of serum triglyceride measure with different triacylglycerol species as a function of (A) the amount, and (B) the number of fatty acid carbons.

Further, the standard serum triglyceride measure correlated best with the most abundant triacylglycerol species in the lipidomics profile, while the correlation was negative for several low abundance triglyceride species (Figure 9A). The total length of fatty acids (i.e. total number of fatty acid carbons) correlated positively with the serum total triglyceride concentration, independently of the degree of fatty acid saturation (Figure 9B).

## Discussion

Environmental and lifestyle factors play a key role in metabolic disorders related to obesity, but it is often very difficult to disentangle these effects from genetic factors that also influence both body fat accumulation and lipid metabolism [10]. Studies of monozygotic (MZ) twins discordant for obesity permit unequivocal distinction between genetic versus environmental and life-style effects. The obese and non-obese MZ co-twins are matched not only for genes but also for age, gender, socioeconomic background and many intra-uterine and childhood environmental factors. The present study focused on young and healthy obesity-discordant MZ twins. We showed that obesity, independent of genetic influences, was related to distinct changes in the global serum lipid profile. In comparison to non-obese co-twins, the obese co-twins had increased levels of lysophosphatidylcholines (LPCs), lipids found in proinflammatory [35], [36] and proatherogenic conditions [37] and decreases in ether phospholipids, which are known to have antioxidant properties [38], [39]. These lipid changes were associated with insulin resistance, a pathogonomic characteristic of the obese co-twins. Our data strongly suggest that obesity-related non-genetic factors make a major contribution to the pre-atherosclerotic and pre-diabetic lipid alterations.

LPCs' proinflammatory and atherogenic properties have been demonstrated in previous studies. These phospholipids upregulate a range of proinflammatory molecules such as P-selectin [40], matrix metalloproteinase-2 [41], cytokines [42], [43], and superoxide anions [44], [45]. LPC increases the permeability of vascular endothelium [46], the process believed to be mediated by a novel subfamily of G protein-coupled receptors (GPR4, G2A, OGR1, and TDAG8) [47]. Increased LPC levels are found in patients with type 2 diabetes [48] and atherosclerosis [49]. In a recent study we have also shown that the LPC levels are elevated in obese Zucker rats at 8 weeks of age relative to lean controls and that PPARγ agonist pioglitazone treatment lowers GPCho(18∶0/0∶0) to levels found in lean Zucker rats [50].

The physiological functions of ether phospholipids are less well understood. There is growing evidence that plasmalogens, the most abundant ether phospholipid subclass characterized by the vinyl-ether bond in sn1 position, can serve as endogenous antioxidants [39]. Plasmalogens may also have a crucial role in states of increased oxidative burden such as hypoxia [51]. Galili and colleagues found recently that increased LPC levels in early obesity in young pigs are associated with endothelial dysfunction and oxidative stress [52]. The combination of high LPC and low ether phospholipid concentration in the present study suggests that LPC and oxidative stress may be linked also in human obesity.

LPCs and ether phospholipids had opposite associations with the M-value, a measure of insulin sensitivity. It is possible that these lipids act as signaling molecules in the insulin cascade. In line with this hypothesis are recent data in rat vascular smooth muscle cells showing that LPC impairs insulin stimulation of insulin receptor substrate (IRS)-1 tyrosine phosphorylation and coupling of the insulin receptor pathway to Akt activation through protein kinase C-α [53]. The role of ether phospholipids in insulin sensitivity remains speculative. The decreases in ether phospholipids in obese co-twins could be due to an increased handling of reactive oxygen species (ROS). High ROS production, rather than low ether phospholipid concentration itself, may then be the factor negatively regulating insulin signaling [54]. Two possible scenarios have been proposed for the lowering of plasmalogens concentrations [55]: (1) Free radical attack to the vinyl-ether bond of plasmalogens, or (2) receptor-mediated degradation by plasmalogen-selective phospholipase A2 (psPLA2). Our data supports the latter scenario since ether linked LPCs such as GPCho(O-20∶3/0∶0), products of PLA2 action, were also found upregulated in obese co-twins (Figure 3).

It is of interest that a docosahexanoic acid (DHA)-containing phosphatidylcholine GPCho(16∶0/22∶6) correlated strongly negatively with the amount of both subcutaneous and intra-abdominal fat (Figure 6) and positively with insulin sensitivity (Figure 7). DHA is an essential n-3 fatty acid with known health benefits [56] but the molecular mechanisms behind its health benefits are poorly understood. Some recent evidence suggests that dietary polyunsaturated fatty acids such as DHA significantly alter the membrane functionality composition of caveolae [57]. Caveolae, specialized membrane microdomains enriched in cholesterol, sphingolipids, and their coat protein caveolin-1, have been implicated in augmentation of insulin signaling [58]. Although found in a variety of cell types, caveolae are most abundant in adipose tissue. Positive correlations of GPCho(16∶0/22∶6) with the M-value and negative correlations with insulin in the present study suggest that DHA containing lipids indeed participate in insulin signaling. Whether the seeming deficiency of serum DHA in obesity reflects the composition of adipose tissue or is related to dietary changes remains to be elucidated.

In addition to many similarities across various fat depots, we also found considerable differences in the lipid profiles associated with various fat depots. Measures of subcutaneous obesity (BMI, total body fat and subcutaneous abdominal fat) correlated with a similar lipid profile whereas intra-abdominal fat had additional specific features. Subcutaneous obesity correlated positively with serum long chain triacylglycerols and negatively with phosphatidylcholine and ether phospholipids within twin pairs. In contrast, intra-abdominal fat correlated positively with medium chain sphingomyelin species. Interestingly, it has recently been demonstrated that adipose sphingolipid metabolism is altered and plasma levels of total sphingomyelin, ceramide, sphingosine, and sphingosine 1-phosphate (S1P) are elevated in genetically obese (ob/ob) mice [59]. Whether the observed changes in the lipid profile and their relationships with adipose tissue distribution can predict insulin resistance and atherosclerosis on an individual basis in humans needs to be studied further.

Obesity did not affect all lipids in serum. Despite the up to 25 kg differences in weight, the very long chain fatty acid (VLCFA) sphingomyelins were very similar in the co-twins (Table 2). Biosynthesis of VLCFA occurs by carbon chain elongation of shorter chain fatty acid precursors. Their β-oxidation, a process controlled by peroxisome proliferator activator receptors (PPARs) [60], takes place almost exclusively in peroxisomes [61]. Our results therefore suggest that the observed proinflammatory changes in early obesity precede the lipotoxic phenomena hallmarked by overflow of saturated fatty acids and dysregulation of the PPAR regulatory system [62].

We also examined how the lipid profile changes reflect differences in classical serum lipid values. Serum triglyceride levels correlated positively with most detected phospholipids and acylglycerols, reflecting the association with the increased flux of these species from the liver in the format of VLDL particles. Interestingly, DHA containing lipids were among the lipids most significantly correlated with classical lipid parameters. This may reflect the rapid incorporation of dietary n-3 fatty acids into the liver nuclei [63] and subsequent release to serum in the lipoprotein particles. Serum LDL concentrations associated positively with LPCs and negatively with ether phospholipids. This supports the proatherosclerotic nature of LDL, especially in its oxidized form. In oxidized LDL, the ratio of LPC to total glycerophosphocholine content increases to 50% from the normal ratio of 1–5% [64]. However, the major carrier of human plasma LPC is albumin [65], demonstrating that the serum global lipid profiles are to an extent unrelated to the classical lipid concentrations.

We might ask why are these MZ twin pairs so strikingly discordant for obesity? Like other MZ pairs in our base population, their growth and weight development was normal in childhood and adolescence. The intra-pair weight differences began to appear only after puberty [17]. This might suggest that the proximate cause(s) of their obesity relates to changes after mid-adolescence, possibly related to differences in their individual life-styles; those differences are not necessarily present after the development of obesity. Therefore prospective studies initiated prior to onset of obesity, preferably in genetically controlled populations, would be needed to conclusively identify environmental causes. It is also possible that the development of obesity is related to an epigenetic modification of gene expression in these MZ pairs. Fraga *et al*. [66] suggested that epigenetic changes increase with increasing age in trait discordant MZ pairs. However, the epigenetic effects relevant to obesity may also develop in childhood or even prenatally. One can speculate that specific environmental factors such as dietary components or sustained physical inactivity could induce development of obesity through modulation of expression of genes regulating satiety [67], as has been shown for social stress and genes affecting depression [65]. Once excess weight development sets in, physical inactivity increases and a vicious circle is ready. What we see in the current analyses are the consequences of this process, but one cannot exclude the possibility that one or more of the lipid species play a causal role in the development of obesity.

One limitation of the present study is the relatively small sample size. Despite a population-based screening of five full birth cohorts of young adult twins, only 14 MZ pairs highly discordant for obesity were found. This is consistent with the evidence that obesity is significantly influenced by genetic factors. Another potential limitation is the fact that the normal weight concordant twins were leaner than twins from other study groups. However, the two main focuses in the study were (a) to compare obese and non-obese twins from discordant pairs and (b) to assess the correlations between intra-pair differences in the clinical measures and those in the lipidomic profiles. Due to a wide range of intra-pair differences in BMI from 0 to 10 kg/m^2^ the subject selection was suitable for this purpose.

In conclusion, our data provide evidence on significant changes of the global serum lipid profile in obesity, independent of genetic influences. Acquired obesity was characterized by increased concentrations of proatherogenic and proinflammatory lysophosphatidylcholine species and decreased concentrations of anti-oxidative ether phospholipids in serum. These lipid changes could account for many of the previously recognized links between obesity and accelerated atherosclerosis. Importantly, the above changes in the global serum lipid profile were associated with insulin resistance, a pathogonomic characteristic of acquired obesity in these presumably healthy young adults. It is likely that proper management of obesity, perhaps with a new generation of therapies directed at several targets in the lipid metabolism pathways, will correct these abnormalities, and favorably modify the risk, course and outcome of diabetes and cardiovascular diseases.

## Supporting Information

Dataset S1 Lipidomics dataset and clinical variables
(0.16 MB XLS)
